# Evidence for using point-of-care diagnostics in the management of respiratory tract infections in primary care: a scoping review protocol

**DOI:** 10.12688/hrbopenres.13770.1

**Published:** 2023-09-25

**Authors:** Judith Cole, Carmel Hughes, Cathal Cadogan, Akke Vellinga, Gerard Molloy, Tom Fahey, Greg Sheaf, Anthony Maher, Cristin Ryan

**Affiliations:** 1 School of Pharmacy and Pharmaceutical Sciences, The University of Dublin, Trinity College, Dublin, Leinster, Ireland; 2 School of Pharmacy, Queen's University Belfast, Belfast, Northern Ireland, UK; 3 School of Public Health, Physiotherapy and Sports Science, University College Dublin, Dublin, Leinster, Ireland; 4 School of Psychology, College of Arts, Social Sciences and Celtic Studies, University of Galway, Galway, County Galway, Ireland; 5 Department of General Practice, Royal College of Surgeons in Ireland, Dublin, Leinster, Ireland; 6 The University of Dublin Trinity College, Dublin, Leinster, Ireland

**Keywords:** Community pharmacists, general practitioners, point-of-care diagnostics, primary care, respiratory tract infections, antimicrobials, antimicrobial resistance

## Abstract

**Background:**

Antimicrobial resistance (AmR) is widely considered a global health threat and is associated with significant morbidity, mortality and costs. Inappropriate antimicrobial use is the most important modifiable risk factor for AmR. Most human antimicrobial medicines use occurs in primary care [prescribed by general practitioners (GPs), dispensed by community pharmacists (CPs)]. However, up to 50% of use is deemed inappropriate. Point-of-care diagnostic tests are used as a basis for antimicrobial stewardship interventions to improve the diagnostic certainty of respiratory tract infections (RTIs), and therefore ensure prudent antimicrobial use. However, there is a lack of guidance on their use, and they are therefore not routinely used in clinical practice.

**Objective:** A scoping review will be conducted to synthesise the available evidence to inform the development of best practice guidance for using point-of-care diagnostics in the management of RTIs in primary care.

**Methods:** A scoping review will be conducted following guidance from the Joanna Briggs Institute (JBI) and reported using the PRISMA-ScR guidelines. Databases including Web of Science, MEDLINE, CINAHL, EMBASE, the International HTA database and the Cochrane Central Register of Controlled Trials, as well as grey literature, will be searched. Screening will be undertaken independently by two reviewers to identify studies and literature reporting the use of point-of-care diagnostics in the management of RTIs in primary care by GPs and/ or CPs. Findings will be described using narrative synthesis.

**Conclusion:**

The findings of this scoping review will be used to produce draft guidance on the use of point-of-care diagnostic tests in primary care, which will undergo further development using a Delphi consensus methodology involving experts in the field of RTIs, antimicrobial stewardship, point-of-care diagnostics and primary care.

## Introduction

Antimicrobial resistance (AmR) is predicted to be the next global health pandemic and is expected to become the leading cause of death by 2050 ^1^. It has been projected by the Organisation for Economic Co-operation and Development (OECD) that over the next 30 years, 2.4 million people throughout Europe, North America and Australia will die because of AmR, and that this will cost up to USD 3.5 billion annually ^2^. One of the main causative but modifiable risk factors associated with AmR is inappropriate antimicrobial use ^3^. The majority (80–90%) of human antimicrobial medicines prescribing takes place in primary care, prescribed by general practitioners (GPs) and dispensed by community pharmacists (CPs). However, up to 50% of this prescribing is deemed inappropriate ^3– 5^. The inappropriate management ( *i.e.* diagnosis and treatment) of respiratory tract infections (RTIs) is particularly problematic, with large variations reported between what is prescribed and what should be prescribed, when the viral aetiologies and self-limiting nature of most RTIs are considered ^6^.

Improving the diagnostic certainly of RTIs in antimicrobial interventions is achieved by the use of point-of-care diagnostic tests ( *i.e.* testing of patients at the time and place of care) ^7^.

Point-of-care tests therefore help to ensure antimicrobials are only prescribed for those who have a bacterial infection, according to the outcomes of the test. Several point-of-care diagnostic tests can be used in the diagnosis of RTIs. Rapid antigen detection tests (RADT) can be used to detect group A *Streptococcus*, and report good sensitivity (86%; 95% CI 83–88) and specificity (96%; CI 94–97) ^8, 9^. A Cochrane review by Cohen and colleagues noted that their use in the management of sore throats in primary care results in reductions (25%) in antibiotic prescribing when compared to clinical management alone ^10^. Point-of-care diagnostic tests involving the detection of inflammatory markers such as C-reactive protein (CRP) and procalcitonin are used in the diagnosis of lower RTI and sepsis, respectively ^11, 12^. A Cochrane review by Tonkin-Crine *et al.* found that point-of-care diagnostics using CRP testing and procalcitonin-guided management significantly reduced antibiotic prescribing for acute RTIs in primary care (RR 0.78 95% CI 0.66–0.92 and OR 0.1 95% CI 0.07–0.14, respectively) ^13^.

The value of using these tests in general practice has also been assessed in health economic evaluations. For example, a study published in 2022 on the cost-effectiveness of CRP point-of-care testing in primary care examined the use of the tests by GPs to guide antibiotic prescribing and found that such prescribing was reduced ^14^. Using a decision-analytic model and data from published studies, CRP POCT was compared to the usual practice of managing patients with RTI symptoms without CRP POCT over five years. The authors reported that CRP point-of-care testing helped to reduce antibiotic prescribing but that healthcare costs were greater, mainly due to the cost of CRP tests. However, they commented that further analysis of a potential CRP point-of-care testing programme over a longer time would be necessary to assess its clinical and cost-effectiveness impact.

Using point-of-care diagnostic tests in the management of RTIs could also have a positive impact on the workload of some primary care healthcare professionals. For example, a United Kingdom (UK) based service was provided by community pharmacists in Wales to test (using RADT to detect group *A Streptococcus)* and treat sore throats ^15^. In total, 11,304 consultations were completed in pharmacies and 8256 (73.0%) of these met the threshold for RADT after a scoring system for sore throat symptoms was applied (FeverPAIN or Centor). Of the patients undertaking a RADT, 2449 (29.6%) tested positive and 2354 (28.5%) were given antibiotics. A total of 410 patients (3.6%) were given a RADT even though they did not meet the scoring criteria, and of these 54 (13.2%) tested positive and 52 (12.7%) were given antibiotics. The authors reported that, had this service not been available in pharmacies, 96% of the service users would have presented to their GP or the emergency setting. Overall, 2406/11,303 (21.3%) patients received antibiotics. This was lower than figures observed from consultations with GPs where rapid antigen detecting is not commonly used (90% of practices prescribe antibiotics for between 35% and 83% of sore throat consultations) ^16^.

Point-of-care diagnostic testing has been found to be accurate. A recent systematic review identified studies which tested the accuracy of a biomarker among patients with acute cough or suspected community acquired pneumonia (CAP) and found that CRP was the most accurate for distinguishing CAP from other causes of lower RTI ^17^. Further, in a cluster randomised controlled trial designed to assess whether point-of-care procalcitonin could reduce antibiotic prescribing in patients with lower RTIs in 60 Swiss general practices, the test was found to result in a 26% absolute reduction in the probability of prescription ^18^.

Despite favourable evidence in support of their use, and the knowledge that point-of-care diagnostic tests are commonly used by patients themselves ( *e.g.* to test for COVID-19), they are not routinely used by GPs or CPs in the management of RTIs in primary care. However, more research is needed to ascertain how such testing should be implemented. This was highlighted by a recent randomised controlled trial which tested the potential for point-of-care CRP testing in respiratory infections in a primary care setting in Vietnam ^19^. The intervention group (24 health centres, n=18,621 patients) received point-of-care CRP testing plus routine care, while the control group (24 health centres, n=21,235) received routine care only. Intention to treat analysis showed that the proportion of patients prescribed antibiotics in the intervention group (93.1%) was lower than the control group (98.2%) (adjusted RR 0·83 [95% CI 0.66–0.93]. While only 2606 patients (14%) in the intervention group had undergone CRP testing, prescriptions were shown to have reduced further in the per-protocol analysis with 1859 patients (73.1%) receiving an antibiotic prescription (adjusted RR *vs* control 0.64 [95% CI 0.60–0.70]). The authors noted that barriers to implementation of CRP testing should be explored and suggested that challenges include difficulties in patients accessing the test (because they were treated remotely), as patients often assumed and expected that they would be prescribed an antibiotic and many were uncertain about having the CRP test.

There is also considerable variation in the use of point-of-care testing and in antibiotic prescribing throughout Europe. A recently published prospective audit of the management of RTIs involving 18 European countries, reported that some countries ( *e.g.* Croatia) did not use any point-of-care diagnostics in the management of RTIs and relied on clinical presentation and opinion alone, while other countries ( *e.g.* Norway) tested for group A streptococcal and/or CRP at point-of-care ^3^. The proportion of patients prescribed antibiotics ranged from 18% in Denmark and Belgium to >50% in Ireland and Hungary. While using point-of-care diagnostics was not associated with lower antibiotic prescribing in this study, the authors concluded that using point-of-care diagnostics may enhance the quality of prescribing decisions made based on clinical grounds alone.

A European expert panel recently published recommendations on the role of CRP point-of-care testing in combatting the overuse of antimicrobials. Recommendations endorse the use of CRP point-of-care testing as being a ‘ *key tool*’ in addressing antimicrobial over-use, but identify that reimbursement structures and the lack of guidelines on the use of CRP point-of-care tests are major barriers to the use of CRP point-of-care testing at national and international levels ^20^. Indeed, there is a lack of guidance on how any point-of-care diagnostic test should be used in the diagnosis of RTIs and the subsequent treatment of patients, either symptomatically or with antibiotics. 

This scoping review therefore aims to synthesise the available evidence on use of point-of-care diagnostics in the management of RTIs in the primary care context to inform the development of best practice guidance for using point-of-care diagnostics in the management ( *i.e.* diagnosis and treatment) of RTIs in primary care.

## Methods

A scoping review was deemed the most suitable methodological approach to ensure that all available evidence regarding the use of point-of-care diagnostics in the management of RTIs in primary care and the recommended treatment strategies based on the outcomes of the point-of-care diagnostic test is retrieved. The Joanna Briggs Institute’s (JBI) approach to conducting scoping reviews has been adopted to ensure methodological rigour ^21^. This approach is based on the framework originally developed by Arskey and O’Malley ^22^, which was further refined by Levac and colleagues ^23^. This framework proposes that the following nine steps should be employed: (1) defining and aligning the objective/s and question/s; (2) developing and aligning the inclusion criteria with the objective/s and question/s; (3) describing the planned approach to evidence searching, selection, data extraction, and presentation of the evidence; (4) searching for the evidence; (5) selecting the evidence; (6) extracting the evidence; (7) analysis of the evidence; (8) presentation of the results and (9) summarising the evidence in relation to the purpose of the review, making conclusions and noting any implications of findings. Steps 1–3 inclusive are described in detail in this protocol. Steps 4–9 will be followed in the reporting of the scoping review. The PRISMA-P checklist has been used in the reporting of this protocol ^24^. The reporting of the review will be guided by the Preferred Reporting Items for Systematic Reviews and Meta-analyses extension for Scoping Reviews (PRISMA-ScR) checklist ^25^. 

### Review question

The research question was developed in line with the Population-Concept-Context (PCC) framework as per JBI recommendations ^21^: What is the available evidence regarding the use of point-of-care diagnostics (concept) in the management of RTIs (population) in primary care (context)? 

## Defining and aligning the objectives and research questions

The overall aim is to summarise the evidence to inform the development of best practice guidance for using point-of-care diagnostic tests in the management ( *i.e.* diagnosis and treatment) of RTIs in primary care, *i.e.* by GPs and/or CPs.

The specific objectives are to:

- Identify the evidence assessing the use of point-of-care diagnostic tests in the management of RTIs in primary care by GPs and/or CPs
- Identify the evidence on diagnostic criteria and clinical prediction rules used in conjunction with individual point-of-care diagnostic tests
- Identify the evidence on treatment strategies based on point-of-care diagnostic test outcomes
- Describe the outcome measures used in studies investigating the effectiveness of point-of-care diagnostic tests for RTIs in primary care
- Identify any gaps in the literature in order to inform the development of best practice guidance for using point-of-care diagnostic tests in the management of RTIs in primary care

## Developing and aligning the inclusion criteria with the objective/s

### Eligibility criteria

The PCC framework ^21^, as described above, will be employed to ensure appropriate study selection, in line with the research question. We will include published national, and international evidence-based guidelines, systematic reviews, quality indicators, grey literature and individual studies that provide guidance on the use of point-of-care diagnostic tests (concept) in the management of RTIs (population) in primary care (context). RCTs, pilot RCT studies and literature providing guidance on the treatment of a subsequently diagnosed RTI in primary care will also be included. Only studies published since January 1, 2010 will be included for pragmatic reasons ( Table 1).

**Table 1.** Eligibility criteria for literature inclusion.

Inclusion criteria	Exclusion criteria
*Date* Studies published since January 1, 2010	Studies published before Jan 2010
*Population* Any patient presenting to a *GP or **CP with suspected ^§^RTI	Patients presenting with non-RTIs
*Concept* Point-of-care diagnostic testing for RTIs	Studies must involve a point-of-care diagnostic test
*Context* Primary care: general practice or community pharmacy	Studies undertaken in a care setting other than primary care *e.g.* the hospital setting
*Types of studies* Systematic reviews ^§§^RCTs and pilot RCTs Published guidelines (national/ international) Grey literature, including policy documents, government documents, theses Quality indicators	Non-English language studies Opinion pieces Case studies Case series Qualitative studies Non-RCTs including cohort and observational studies, and controlled before-and-after studies

*GP: General Practitioner **CP: Community pharmacist ^§^ RTI: Respiratory Tract Infection ^§§^RCTs: Randomised Controlled Trials

### Population

The eligible population for this review includes patients presenting to GPs or CPs in primary care with a suspected RTI. No age restrictions apply.

### Concept

This scoping review will consider any point-of-care diagnostic test used to diagnose any RTI.

### Context

This review will include literature that provides guidance on the use of point-of-care diagnostic tests for the management of suspected RTIs in primary care, and the subsequent treatment of diagnosed RTIs in primary care. Literature that does not focus on the primary care healthcare setting will not be included.

## The planned approach to evidence searching, selection, data extraction, and presentation of the evidence

### Searching the evidence

An initial limited search of Web of Science was undertaken to identify relevant articles, using truncated keywords and MeSH terms provided in Table 2, based on the PCC framework, such as ‘primary care, ‘point-of-care’, ‘respiratory tract infection’. This was undertaken so that index terms used to describe these relevant articles and their abstracts could be used to develop the full search strategy. An initial search strategy has been completed for Web of Science, in conjunction with a specialist subject librarian (GS) and is provided in Table 3. This search strategy will be adapted for other databases including MEDLINE, CINAHL, EMBASE, the International HTA Database and the Cochrane Central Register of Controlled Trials. Grey literature searching will be undertaken, *e.g.*, policy literature and government documents, using databases such as OpenGrey ( www.opengrey.eu) and Scopus.

**Table 2.** Relevant Population Concept Context (PCC) search terms with truncated keywords and MeSH terms for Web of Science .

	Population	Concept	Context
Definition	Any patient with a suspected RTI *	Point-of-care diagnostic test for the suspected RTI	Primary care
Web of Science (MeSH-terms)	Respiratory tract infections RTI/s Infection Chest Common cold Sinusitis Pharyngitis Tonsillitis Rhinitis Laryngitis Bronchitis Croup Epiglottitis Respiratory tract disease Pleurisy Pneumonia Influenza Cough Sneeze Sore throat Runny nose Congestion Headache Earache Otalgia Muscle ache Breathless Wheeze High temperature	Point-of-care Near-patient Procalcitonin C-reactive protein CRP Biomarker	Primary care Family doctor General physician General practitioner Community pharmacist Ambulatory

*RTI: Respiratory Tract Infection

**Table 3.** Search strategy for Web of Science.

Language	English-Only
Search terms relating to population (separated by Boolean operator ’OR’)	(((Respiratory OR chest) NEAR/2 (disease* OR infection*)) OR “RTI*” OR Bronchitis OR “Common cold*” OR Croup OR Epiglottitis OR Flu OR Influenza* OR Laryngitis OR Laryngotracheitis OR Otalgia OR Pharyngitis OR Pleurisy OR Pneumo* OR Rhinitis OR Sinusitis OR Tonsillitis OR Breathless* OR Congestion OR Cough* OR Earache* OR Headache* OR “High temperature*” OR “Muscle ache*” OR “Runny nose*” OR Sneez* OR “Sore throat*” OR Wheez*)
Boolean operator	AND
Search terms relating to context ( separated by Boolean operator ’OR’)	(((Family OR General) NEAR/2 (doctor* OR physician* OR pract*)) OR ((Community OR Primary) NEAR/2 (care OR center* OR centre* OR healthcare)) OR Ambulatory OR Pharmac* OR (Home NEAR/2 visit*))
Boolean operator	AND
Search terms relating to concept ( separated by Boolean operator ’OR’)	(“Point of care” OR “POCT” OR diagnostic* OR Procalcitonin OR “C-reactive protein*" OR “CRP” OR Biomarker*)

### Selection of evidence

All titles and abstracts retrieved from searches will be transferred into the online platform, Covidence ^®^^26^, to manage each step of the review process. All duplicates will be removed. All titles will be screened for eligibility based on inclusion and exclusion criteria ( Table 1), followed by abstracts and then full texts. Two review authors (JC, CR) will undertake these reviews independently and resolve any disagreement by discussion with a third reviewer. Reasons for exclusion at full-text review will be noted. 

### Data extraction

Data extraction will be undertaken by two reviewers independently, using a data extraction form developed and piloted by both reviewers prior to commencement of this process. A draft data extraction table is provided in Table 4. Any modifications made to this tool throughout data extraction will be detailed in the final scoping review report. Data relevant to the research questions will be extracted, including a description of the point-of-care diagnostic tests used in the management of RTIs in primary care, when they are used, which healthcare professional in primary care uses them, the communication amongst the primary care-based healthcare team and patients, for example, communication of diagnoses and prescribing decisions between GPs and pharmacists, how diagnosed RTIs are treated and what symptomatic management is provided to those who do not have a RTI. If necessary, authors of included literature will be contacted to request missing data. Disagreements between review authors will be resolved by discussions and will involve a third review author if necessary.

**Table 4.** Data extraction form.

Author, Year, Country	Aim	Type of evidence source ( *e.g*. research report/ policy document)	Study design	Population (Description)	Concept (Type of *POC test)	Context ( ^§^HCPs involved)	Description of POC test use ( *e.g.* type, when, where, by whom)	Description of additional diagnostic criteria used	Description of communication of *POC test outcome within primary healthcare team ( *e.g.* between GP and CP). What was communicated and how ( *e.g.* electronic / telephone)	Description of outcomes measured	Description of treatment provided (e.g. antibiotic/ symptomatic management)
											
											

*POC: Point-of-care ^§^HCP: Healthcare professional

### Assessment of the quality of included studies

As the aim of scoping reviews is to provide a broad overview of existing literature, assessment of methodological quality of included studies is not routinely undertaken ^23^. However, for the current scoping review, we will critically appraise the included studies using the appropriate JBI Critical Appraisal Checklist ^27^. This assessment will be completed independently by two reviewers (JC and CR). Disagreements will be resolved by discussion with a third reviewer, if necessary. The quality of evidence stemming from existing guidelines will be assessed using the Appraisal of Guidelines for Research and Evaluation (AGREE II) assessment tool ^28^.

### Presentation of the evidence

**Figure 1.**
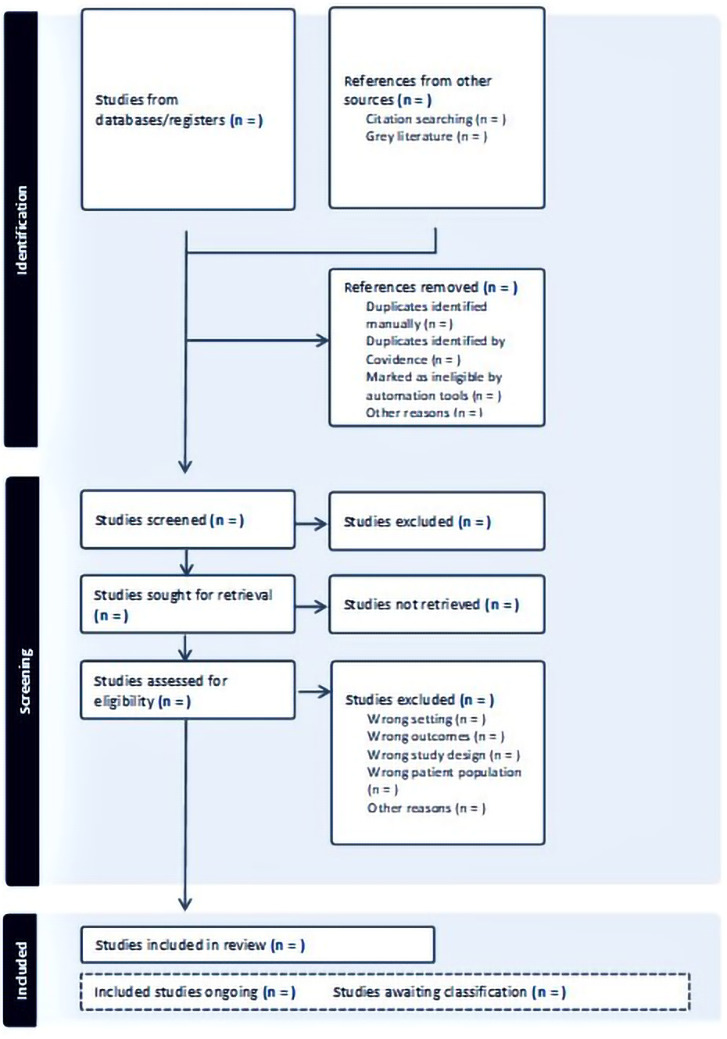
PRISMA flow diagram for scoping review: Evidence for using point-of-care diagnostics in the management of respiratory tract infections in primary care.

The search results will be reported in the final scoping review and presented in a Preferred Reporting Items for Systematic Reviews and Meta-analyses extension for scoping review (PRISMA-ScR) flow diagram ( Figure 1). Key findings will be presented in tabular format, which will be developed and refined throughout the data extraction process. The findings will be synthesised in a narrative format according to scoping review guidelines. The narrative summaries will be presented along with the tables and provide an overview of the evidence for each point-of-care diagnostic test, the healthcare professional using the point-of-care diagnostic test and treatment strategies based on the outcomes of each test. Gaps in the research evidence will also be identified.

## Study status

The scoping review is currently at the full-text screening stage. A total of 13,201 studies were sourced from database searches. Of these, 3161 duplicates were removed leaving 10,040 titles for screening. After 9824 studies were deemed irrelevant, 216 full-text articles were passed to the full-text screening stage and when this is completed data extraction will begin.

## Discussion

Using point-of-care diagnostic tests in the management of RTIs could serve as an important antimicrobial stewardship strategy to improve the appropriate management of RTIs and encourage more prudent use of antimicrobials in primary care. The lack of guidance to support their use has been identified as one of the barriers to their widespread use ^20^. 

The findings of this scoping review will be used to produce draft guidance on using point-of-care diagnostics tests in the management of RTIs in primary care. The content of the draft guidance will include details on what point-of-care diagnostic tests should be performed (depending on symptomatology), who should provide the test, *i.e.* GPs/CPs/both, where the test should be provided (general practice/community pharmacy/both) and how and when outcomes of tests should be communicated within the primary healthcare team, *i.e.* between GPs and CPs and with patients. Guidance on the appropriate management, including when antibiotics are indicated and guidance on patient self-management, will also be included. This initial draft guidance will undergo a Delphi validation ^29^ process, where experts in the field of RTIs, antimicrobial stewardship, point-of-care diagnostics and primary care, *i.e.* GPs and CPs, will be asked to agree on the content of the guidance. Following agreement, views of GPs, CPs and patients will be sought to establish the perceived barriers and facilitators to using point-of-care diagnostic tests in the management of RTIs.

## Data Availability

No data are associated with this article.
