# Supplementary material for: Evidence for using point-of-care diagnostics in the management of respiratory tract infections in primary care: a scoping review protocol
Source: HRB Open Res. 2023 Sep 25;6:59. [Version 1] doi: 10.12688/hrbopenres.13770.1 (PMC10822039; doi:10.12688/hrbopenres.13770.1)
Supplement: Peer review history

# Reviewer report: Reviewer response for version 1

**DOI:** 10.21956/hrbopenres.15062.r37738

**Published:** 2024-01-27

**Authors:** Sara Haddad

**Affiliations:** 1 American University of Beirut Medical Center, Beirut, Lebanon

The article addresses the pressing issue of antimicrobial resistance and its association with inappropriate antimicrobial use, which is a significant global health concern. The majority of antimicrobial prescribing occurs in primary care settings, where general practitioners prescribe and community pharmacists dispense medications. However, up to 50% of this prescribing is deemed inappropriate, especially in cases of respiratory tract infections where antibiotics may be prescribed unnecessarily. To improve the appropriate use of antimicrobials, the article proposes the use of point-of-care diagnostic tests in primary care. These tests, which can be performed at the time and place of care, aim to enhance diagnostic certainty for RTIs and guide antibiotic prescriptions. Various point-of-care diagnostic tests, including Rapid Antigen Detection Tests, CRP, and procalcitonin, have shown promise in reducing antibiotic prescribing. Despite evidence supporting their effectiveness, point-of-care diagnostic tests are not routinely used. To address this gap, the article outlines a scoping review protocol. The scoping review aims to synthesize existing evidence on the use of point-of-care diagnostics in primary care settings to inform the development of best practice guidance. The study aims to identify evidence related to the use of point-of-care diagnostic tests, diagnostic criteria, treatment strategies based on test outcomes, and outcome measures used in primary care, and to produce draft guidance on the use of point-of-care diagnostic tests in primary care.

Is the study design appropriate for the research question?

Yes

Is the rationale for, and objectives of, the study clearly described?

Yes

Are sufficient details of the methods provided to allow replication by others?

Yes

Are the datasets clearly presented in a useable and accessible format?

Yes

Reviewer Expertise:

Infectious diseases

I confirm that I have read this submission and believe that I have an appropriate level of expertise to confirm that it is of an acceptable scientific standard.

# Reviewer report: Reviewer response for version 1

**DOI:** 10.21956/hrbopenres.15062.r36536

**Published:** 2023-11-03

**Authors:** Noémie Boillat-Blanco, Cécile Bessat

**Affiliations:** 1 Infectious Diseases Service, University Hospital of Lausanne, University of Lausanne, Lausanne, Switzerland

**General comment:**

 We read with much attention and interest the article “Evidence for using point-of-care diagnostics in the management of respiratory tract infections in primary care: a scoping review protocol”. This is a well written and comprehensive protocol for a scoping review on the evidence behind the use of point of care test for respiratory tract infection (RTI) in primary care.

 We congratulate the authors for undertaking this meaningful work. They identified a gap between existing published evidence on the use and impact of POCT in primary care and their absence in the guidelines. Their work will provide an extensive overview of the evidence behind POCT use and impact in primary care. These data will be used to produce guidance on the use of POCT in primary care, further adapted using a Delphi consensus involving experts. This work participates in bridging the gap between evidence and implementation in primary care.

**Rational and objectives:**

 The rational for the review is well described: point-of-care test are available to enhance the diagnostic accuracy of RTIs and thereby promote responsible use of antimicrobial drugs, but there is a current lack of guidance which is leading to a limited integration into primary care clinicalpractice. The objectives are therefore to summarize the existing evidence on the use of point-of-care test for the diagnosis and management of RTI in primary careand contribute to the establishment of guidelines.

 The authors chose to include all respiratory tract infections: 1) sore throat (clinical suspicion of streptococcal pharyngitis) and 2) cough (clinical suspicion of pneumonia). Could the author justify why they include both clinical entities in the same work as the type of POCT will be very different?

**Design**

 Regarding the design - a scoping review – the authors state that “it was deemed the most suitable methodological approach to ensure that all available evidence is retrieved”. Could they explain more in details why they did not choose to do a systematic review? We understand it will provide a mapping of the available evidence with a broader scope than a systematic review. A paragraph detailing the reasons behind their choice would be useful.

**Methods**

 We have a few comments on the methods:

- Could the author specify better why they chose only to include studies published from 2010, an whether this could lead to missing evidence?
- Have they thought about looking at ongoing or unpublished clinical trial in ClinicalTrials.gov, clinicaltrialsregister.eu and the International Clinical Trials Registry Platform of the World Health Organization?
- The authors will include different types of studies in the review (Systematic reviews, RCTs and pilot RCTs, Published guidelines, Grey literature, policy documents, government documents) that are highly heterogeneous in terms of population, methodology and objectives. How will they describe the strength of evidence in the review? (as systematic reviews often do using tools like the GRADE system).

**Conclusion**

 We are looking forward to reading the future scoping review when it will be available

Is the study design appropriate for the research question?

Yes

Is the rationale for, and objectives of, the study clearly described?

Yes

Are sufficient details of the methods provided to allow replication by others?

Yes

Are the datasets clearly presented in a useable and accessible format?

Not applicable

Reviewer Expertise:

NA

We confirm that we have read this submission and believe that we have an appropriate level of expertise to confirm that it is of an acceptable scientific standard.
